# Dietary Phytochemicals Targeting Nrf2 to Enhance the Radiosensitivity of Cancer

**DOI:** 10.1155/2022/7848811

**Published:** 2022-03-23

**Authors:** Pinghan Wang, Fangyi Long, Hong Lin, Song Wang, Ting Wang

**Affiliations:** ^1^Laboratory Medicine Center, Sichuan Provincial Maternity and Child Health Care Hospital, Affiliated Women's and Children's Hospital of Chengdu Medical College, Chengdu Medical College, Chengdu 610041, China; ^2^Department of Pharmacy, Sichuan Cancer Hospital & Institution, Sichuan Cancer Center, School of Medicine, University of Electronic Science and Technology of China, Chengdu 610041, China

## Abstract

Nowadays, cancer has become the second leading cause of death worldwide. Radiotherapy (RT) is the mainstay in management of carcinoma; however, overcoming radioresistance remains a great challenge to successfully treat cancer. Nrf2 is a key transcription factor that is responsible for maintaining cellular redox homeostasis. Activation of Nrf2 signaling pathway could upregulate multifarious antioxidant and detoxifying enzymes, further scavenging excessive reactive oxygen species (ROS). Despite its cytoprotective roles in normal cells, it could also alleviate oxidative stress and DNA damage caused by RT in cancer cells, thus promoting cancer cell survival. Accumulating evidence indicates that overactivation of Nrf2 is associated with radioresistance; therefore, targeting Nrf2 is a promising strategy to enhance radiosensitivity. Dietary phytochemicals coming from natural products are characterized by low cost, low toxicity, and general availability. Numerous phytochemicals are reported to regulate Nrf2 and intensify the killing capability of RT through diverse mechanisms, including promoting oxidative stress, proapoptosis, and proautophagy as well as inhibiting Nrf2-mediated cytoprotective genes expression. This review summarizes recent advances in radiosensitizing effects of dietary phytochemicals by targeting Nrf2 and discusses the underlying mechanisms, including N6-methyladenosine (m6A) modification of Nrf2 mediated by phytochemicals in cancer.

## 1. Introduction

Currently cancer is the second leading cause of death worldwide [1], and it is also the most important risk factor of mortality in 112 countries estimated by the World Health Organization (WHO) [2]. The data from GLOBOCAN 2020 showed that new cases and deaths of cancer worldwide have exceeded 19.3 million and 9.9 million, respectively [3]. Obviously, the rapidly increasing burden of cancer throughout the world is alarming [4], and it is extremely urgent for us to utilize various strategies to combat cancer.

Radiotherapy (RT) is a mainstay of cancer therapy, which is used alone or in combination with chemotherapy, surgery, and immunotherapy by cancer patients [5]. And RT was conducted with multiple ionizing radiation includes *α*- or *β*-particles, X-rays, *γ*-rays, and neutron beams [6]. RT might inhibit the growth of tumor cells effectively by generating high levels of reactive oxygen species (ROS) via water radiolysis, which further destroy cellular materials including lipids, proteins, and DNA [7]. However, some cancer cells could alleviate the detrimental effects induced by radiation through enhancing redox and antioxidant defenses, thus resulting in radioresistance [8, 9]. Furthermore, the change of micro-environment in radioresistant cells would further restrict the efficacy of RT and eventually promote tumor metastasis and recurrence [10]. Therefore, it is significantly crucial to overcome radioresistance of cancer cells to improve the therapeutic efficacy.

Accumulating scientific evidence suggests that the elevated levels of antioxidants prevent cancer cells from radiation-induced damages; therefore, blocking these defense networks would restore their response to radiation [11–13]. NF-E2-related factor 2 (Nrf2) is a predominant transcription factor that regulates the expression of antioxidant enzymes. It contains a basic-region leucine zipper (bZIP) DNA-binding domain, which could bind to the promoter region of antioxidant responsive element/electrophile responsive element (ARE/EpRE) and induce cytoprotective downstream genes including antioxidant enzymes [14]. Thus, suppressing Nrf2 might become a promising strategy to increase the radiosensitivity of cancer cells. Despite the fact that Nrf2 regulators are not currently in clinical use, however, it has been shown that some dietary phytochemicals could resensitize tumor cells to RT by inhibiting Nrf2 in some preclinical studies [15, 16]. In this review, we aim to summarize the dietary phytochemicals targeting Nrf2 to increase the radiosensitivity of tumor cells and also provide novel insights on the potential epigenetic regulation of Nrf2 by the phytochemicals.

## 2. Nrf2/ARE Signaling Pathway

**Figure 1:**
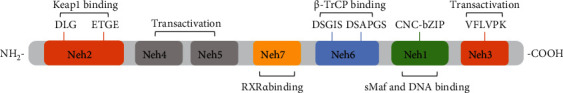
Domain structure of Nrf2 protein.

Nrf2 (Figure 1) is a 66 KD protein encoded by NFE2L2 gene, and it is a key transcription regulatory factor that plays important roles in the maintenance of the cellular redox homeostasis [17, 18]. Nrf2 has seven highly conserved functional domains, called Nrf2-ECH homology (Neh 1-7) [18]. Among these functional domains, Neh2 was located in the N-terminal, which contains ETGE and DLG motifs for binding the inhibitory protein Kelch-like ECH-associated protein 1 (Keap1) [19–21]. Neh1, with a cap “n” collar (CNC)-type bZIP motif, allows the binding of Nrf2 to ARE/EpRE by regulating the heterodimerization of Nrf2 with small musculoaponeurotic fibrosarcomas (sMaf) family, such as MafF, MafG, or MafK [22–24]. The C-terminal Neh3 domain possesses a VFLVPK motif, which is crucial for interactions with CHD6, the transcription co-activator of Nrf2, to mediate the transactivation of ARE-dependent genes [25, 26]. Neh4 and Neh5 domains act synergistically with Nrf2 and responsible for ARE transactivation by interacting with the coactivator CBP (CREB-binding protein) [24, 27]. The redox-insensitive Neh6 domain is riched in serine residues and contains two motifs (DSGIS and DSAPGS) which could negatively modulate Nrf2 stability through *β*-TrCP-dependent regulation [28–30]. Neh7 domain could directly bind to the retinoic X receptor *α* (RXR*α*) and further transcriptionally inhibit Nrf2 target genes [31].

**Figure 2:**
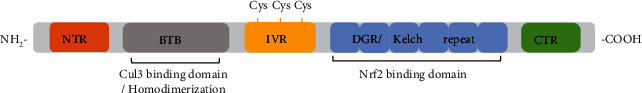
Domain structure of Keap1 protein.

Keap1 (Figure 2), a cysteine-rich and highly conserved protein encoded by the KEAP1 gene, was first identified in 1999 as negative regulator of Nrf2 by binding to the Neh2 domain [21, 32]. As a substrate adaptor for Cullin3 (Cul3)-based ubiquitin E3 ligase, Keap1 could contribute to Nrf2 ubiquitination and subsequent proteasome-dependent degradation [33, 34]. Structurally, Keap1 is composed of five distinct functional regions: an N-terminal region (NTR), a broad-complex, Tramtrack and Bric-a-brac (BTB) domain, an intervening region (IVR), a double-glycine repeat (DGR, namely, six Kelch motifs) domain, and the C-terminal region (CTR) [35]. The BTB domain is responsible for both Keap1 homodimerization and the recruitment of Cul3 protein [36]. The cysteine-rich IVR region could interact with Cul3-Roc1-E3 ubiquitin ligases complex and regulate the activity of Keap1 [37, 38]. The DGR domain could recognize the ETGE and DLG motifs within the Neh2 region of Nrf2, enabling ubiquitination and proteasomal degradation [29, 39].

**Figure 3:**
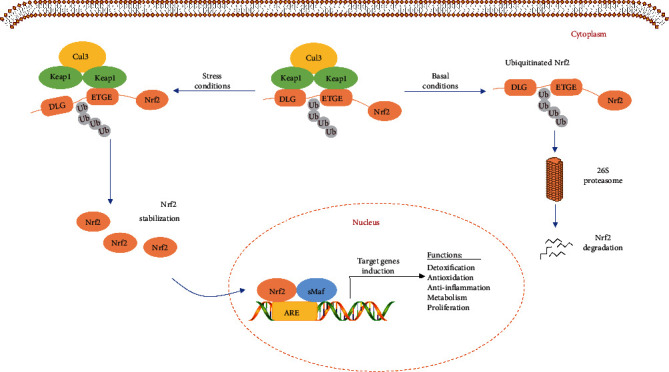
Schematic representation of the Keap1-Nrf2-ARE pathway. Under basal condition, the DLG and ETGE motifs of Nrf2 interact with the BTB domain contained within Keap1, which brings Nrf2 to the Cul3-based ubiquitin E3 ligase and targets the 26S proteasome for degradation in cytoplasm. Under stressed condition, the modified cysteine residues disturb the binding between Keap1 and Cul3, which protect Nrf2 from proteasomal degradation. The accumulated Nrf2 subsequently translocates into the nucleus, dimerizes with sMaf, and binds to the ARE which is located in the promoter of the target cytoprotective genes, ultimately driving their robust expression.

Keap1-Nrf2-ARE signaling acts as master pathway for the maintenance of cellular redox state [40]. As depicted in Figure 3, under physiological state conditions, Nrf2 is sequestered in the cytosol by Keap1, which constitutively brings Nrf2 to the Cul3-RBx1-E3 ubiquitin ligase and targets Nrf2 to proteasomal degradation [41–43]. Under oxidative stress, Keap1 is inactivated due to the modified cysteine residues, leading to the accumulation and nuclear translocation of the newly synthesized Nrf2, which forms a heterodimer with sMaf and interacts with the ARE to transcribe its target genes including heme oxygenase-1 (HO-1), NAD[P]H:quinone oxidoreductase 1 (NQO1), glutathione reductase (GR), and superoxide dismutase (SOD), thus protecting cells from oxidative damages [38, 41, 44].

Moreover, phosphorylation of Nrf2 Serine 550 residue mediated by adenosine 5′-monophosphate-activated protein kinase (AMPK) is essential for the nuclear translocation of Nrf2 [45]. Additionally, there are other pathways leading to this process; for example, Nrf2 could also be activated via the Akt-mediated inhibition of glycogen synthase kinase 3 beta (GSK-3*β*) [46]. Furthermore, various posttranslational modification (PTM)- related proteins are involved in the dissociation of Nrf2 from Keap1, such as extracellular signal-regulated kinase (ERK) and c-Jun NH2-terminal protein kinase (JNK), hence resulting in the activation of Nrf2 [46]. And certain factors like p21, p62, or breast cancer gene 1 (BRCA1) facilitate the stabilization of Nrf2 through inhibiting its ubiquitination and degradation, accomplished by interfering with the interaction of Keap1 and Nrf2 [46, 47]. Despite these regulations confer cytoprotective effects in normal cells, interestingly, the upregulation of antioxidant enzymes by abundant accumulation of Nrf2 might induce cell growth or enhance the resistance to chemotherapy and radiotherapy in cancer cells [47, 48].

## 3. Role of Nrf2 in Cancer Radioresistance

Nowadays, RT is a commonly accepted and effective approach to treat various types of malignancies [49]. The basic principle of RT to eradicate cancer cells is to directly destroy single- or double-strand nucleic acid molecules, or indirectly lead to DNA breaks via free radicals, largely by means of ROS production (such as O_2_^−^, H_2_O_2_, and OH^−^), and ultimately induce cells necrosis or apoptosis [6, 50]. Meanwhile, an increasing number of studies demonstrated that the accumulation of ROS plays a crucial role in RT by damaging DNA biomolecules and triggering cell death-related signaling pathways [51–54]. Under physiological circumstance, intracellular ROS is kept at a relatively low level and is precisely controlled by the scavengers, such as glutathione, thioredoxin, peroxidase, and catalase [55]. However, the increased ROS scavenging substances levels or the enhancements of antioxidant defense systems might contribute to radioresistance in cancer cells [56, 57]. Radioresistance is the process by which the tumor cells or tissues adapt to RT and develop resistance to it [58]. Despite significant therapeutic achievements in recent years, radioresistance still hinders tumor loco-regional control and promotes tumor progression in most patients [59, 60]. The mechanisms underlying cancer cell radioresistance usually involve several factors as below: enhanced DNA repair capabilities, activated cell cycle control proteins, dysregulation of oncogenes and tumor suppressors, changes in the tumor microenvironment (TME), dysfunctional autophagy, epithelial-to-mesenchymal transition (EMT), generation of cancer stem cells (CSCs), tumor metabolism alterations, and activation of intracellular ROS scavenging [55, 58]. In addition, a growing number of studies have shown that upregulated expression of antioxidant enzymes contributes lower cellular ROS levels and leads to poor response to RT, while impeding these ROS-elimination systems, such as the Nrf2 pathway, could increase radiation sensitivity and promote radiation-mediated apoptosis [61–64].

**Figure 4:**
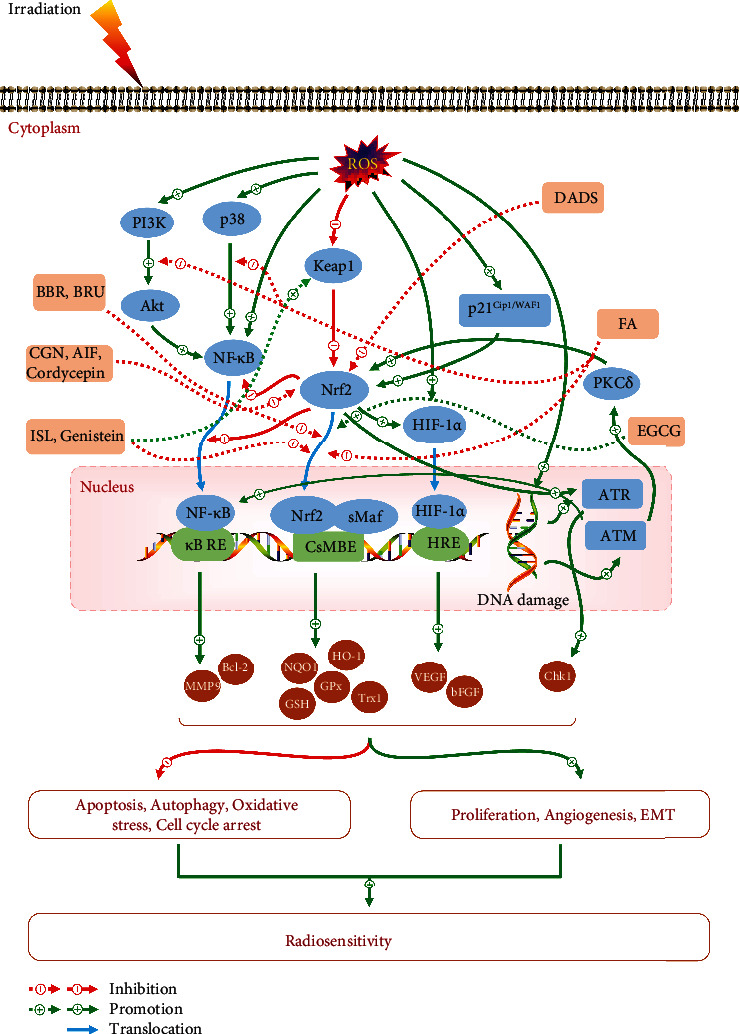
The signaling pathways involved in Nrf2-mediated radioresistance and the potential targets of dietary phytochemicals. Radiation produces abundant ROS, leading to the activation of signaling pathways including Nrf2, p38 MAPK, and Akt/PI3K. Upregulation of Nrf2 would further elevate the levels of antioxidants, such as HO-1, NQO1, Trx1, GPx, and GSH in cancer cells, ultimately leading to tumor radioresistance. Nrf2 has also been implicated in the roles of other genes in cancer radioresistance via interactions with NF-*κ*B, HIF-1*α*, ATM, ATR, and p21^Cip1/WAF1^. Among these dietary phytochemicals, EGCG is capable to enhance the killing effects of RT by promoting Nrf2 expression; however, other phytochemicals, such as BBR, BRU, CGN, AIF, ISL, DADS, FA, genistein, and cordycepin, have been shown to enhance radiosensitivity through inhibiting Nrf2 expression, or suppressing Nrf2 nuclear translocation, Akt/PI3K/NF-*κ*B pathway, and p38 MAPK/NF-*κ*B pathway, or promoting Keap1 expression.

Persistent expression of Nrf2, a status termed “Nrf2 addiction,” may coordinate the positive regulation of multiple hallmarks of cancer, including promotion of metastasis and metabolic changes [65, 66]. There is substantial preclinical data implicated in Nrf2 addiction for various causes, for instance, (1) the genetic and epigenetic changes of Keap1 or Nrf2 or noncoding RNAs modulation; (2) the activation of oncogenic signaling such as K-Ras and c-Myc; (3) stress stimulation such as hypoxia, starvation, and genotoxic stress; (4) altered protein-protein interactions, such as the p62-Keap1 or p21-Nrf2 linkage, and the posttranslational dysregulation of the Keap1-Nrf2 pathway; and (5) ROS-inducing gonadotropins and sex steroid hormones [43, 67]. Indeed, hyperactivation of Nrf2 drives malignant phenotype of cancers by significantly increasing resistance to chemo- and radiotherapy and also promotes the aggressive development of many different types of tumors [65]. This phenomenon was described by Wang et al. as a “dark side” of Nrf2 [68]. Moreover, fractionated or single doses of ionizing radiation could activate Nrf2 by elevating ROS levels [69, 70], which further conferring enhanced target genes expression, including HO-1, NQO1, peroxiredoxin 1, and glutathione [48, 71, 72]. Nrf2 also interacts with some signaling networks associated with radioresistance, such as hypoxia-inducible factor 1 (HIF-1) and nuclear factor-kappa B (NF-*κ*B) to enhance the resistance against RT [48, 71]. To date, several studies have reported that increased activity of Nrf2 results in radioresistance, while inhibiting Nrf2 expression resensitizes cancer cells to RT, including lung [73, 74], esophageal [72, 75], breast [76], and prostate carcinoma [77]. These findings indicate that Nrf2 is critical in mediating radioresistance against RT, and researchers have paid considerable attention to target Nrf2 in tumors by chemical inhibitors, especially dietary phytochemicals, which have the ability to sensitize cancer cells to RT [71]. The impacts of Nrf2 on radiation resistance are described in Figure 4.

## 4. Targeting Nrf2 to Enhance the Radiosensitivity by Dietary Phytochemicals

Dozens of phytochemicals have been reported to modulate Nrf2 to exert the preventive or therapeutic effects in various cancers [78, 79]. Natural Nrf2 activators are mainly a class of electrophilic or redox-active compounds, such as sulforaphane, curcumin, andrographolide, resveratrol, and quercetin, which could covalently modify the cysteine residues of Keap1 by oxidation or alkylation, thus stabilizing Nrf2 [79, 80], and they play antitumor or chemopreventive roles in tumourigenesis [81]. Nevertheless, the natural Nrf2 inhibitors, like halofuginone, luteolin, wogonin, trigonelline, malabaricone A, and ascorbic acid, could function as anti-carcinogens or chemosensitizers in tumors via reducing Nrf2 generation or its nuclear localization [79, 82]. Interestingly, there are some opposing studies, which claimed that luteolin [83] and wogonin [84] could activate Nrf2 pathway, while curcumin [85] could negatively regulate Nrf2. Therefore, future studies may focus on exploring the dual functions of these natural products to develop more applications for Nrf2.

**Figure 5:**
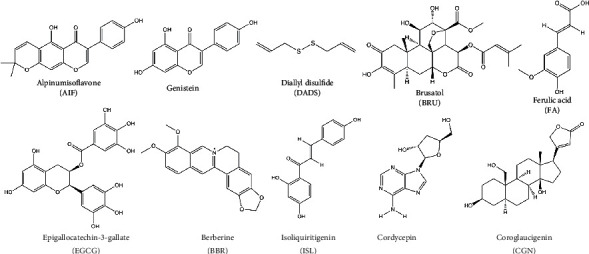
Chemical structures of dietary phytochemical targeting Nrf2 that could potentially enhance radiosensitivity.

As aberrant regulation of Nrf2 protects cancer cells from RT injury, pharmacological modulation of the Nrf2 pathway offers novel therapeutic opportunities to reduce radioresistance. A large number of natural compounds included in our dietary spectrums have been identified as Nrf2 inhibitors [86]. Here, citations for this review were searched and selected from the PubMed and Google Scholar (from January 1989 to January 2022). Experimental papers on natural radiosensitizers and Nrf2 as therapeutic mechanisms for these compounds were identified. The keywords used in the literature research were “Nrf2” or “Nrf-2,” “NFE2L2” or “nuclear factor erythroid 2-related factor 2,” in combination with “radiosensitivity,” “radiotherapy,” “radioresistance” or “radiosensitizer.” Only literatures involving the above inclusion criteria were then manually screened. Here, the bioactive phytochemicals currently available for RT radiosensitization via Nrf2 inhibition mechanisms are summarized below (Table 1). Their structures are presented in Figure 5. The mechanisms by which these Nrf2 modulators mitigates radioresistance are illustrated in Figure 4.

### 4.1. Isoliquiritigenin (ISL)

ISL ((E)-1-(2,4-dihydroxyphenyl)-3-(4-hydroxyphenyl)prop-2-en-1-one), a flavonoid with a chalcone structure, is mainly isolated from the roots of the plant licorice, which generally known as *Glycyrrhiza*, including *Glycyrrhiza uralensis*, *Glycyrrhiza radix*, and *Glycyrrhiza glabra* [97]. ISL displays a wide range of potent biological functions and pharmacological effects, such as antioxidation, antitumor, antiaging, anti-inflammation, and antidiabetes [98]. Recently, Sun et al. showed that ISL substantially sensitized HepG2 cells to 4 Gy X-rays after pretreatment with low concentration of ISL for 6 h by using cells and xenograft models [87]. It was also found that the effectiveness of ISL was associated with inhibition of HepG2 cell proliferation and increased DNA impairment and apoptosis, mainly due to Keap1-dependent downregulation of Nrf2 and elevating NADPH oxidase 2 (Nox2) expression [87, 88]. However, despite these advantages, ISL was also reported to cause developmental toxicity in zebrafish pups [99].

### 4.2. Genistein

Genistein (5,7-dihydroxy-3-(4-hydroxyphenyl)chromen-4-one) is nontoxic isoflavone and phytoestrogen, mainly derived from soybean and with high bioavailability and low water solubility. It exhibits various biological and pharmacological activities, such as antitumor, proapoptotic, antiproliferative, tyrosine- and topoisomerase-inhibiting, and anti-osteoporotic effects [100, 101]. Genistein is also found in other edible plants such as alfalfa, broccoli, sunflower, caraway, and clover seeds [101]. In recent years, a large quantity of studies have pinpointed that genistein has radiosensitizing effects on various tumors including liver cancer, mammary tumor, lung cancer, and leukemia through multiple mechanisms [11, 89, 102, 103]. Specifically, in A549 cancer cells, 10 *μ*M genistein combined with 4 Gy X-ray irradiation markedly promoted cellular oxidative damage and apoptosis by restraining CpG island methylation of the Keap1 promoter sequence and reducing nuclear translocation of Nrf2 along with its downstream targets NQO1, HO-1, and GSH, to alleviate radioresistance [89].

### 4.3. Coroglaucigenin (CGN)

Cardenolides, a subclass of cardiac glycosides (CGs), are widely distributed in numerous long-term cardioprotective medicinal plants with steroid-like structures and potential anti-tumor activities [104]. CGN (3-[(3S,5S,8R,9S,10R,13R,14S,17R)-3,14-dihydroxy-10-(hydroxymethyl)-13-methyl-1,2,3,4,5,6,7,8,9,11,12,15,16,17-tetradecahydrocyclopenta[a]phenanthren-17-yl]-2H-furan-5-one), belonging to cardenolides, derived from the roots or stems of plant *Calotropis gigantea*, and has significant cytotoxicity against several cancers including liver, gastric, colorectal and lung cancer in vitro [90, 105]. Recently, Sun et al. demonstrated that physiologically achievable low doses of CGN were less toxic to BEAS-2B cells and had the potential to enhance X-rays lethality in 3 human lung cancer cells (A549, NCI-H460 and NCI-H446) via exacerbating anticipated oxidative stress and radiation-induced DNA oxidative damage. These effects are associated with repressive activation of Nrf2 and downstream proteins [90].

### 4.4. Cordycepin

Cordycepin ((2R,3R,5S)-2-(6-aminopurin-9-yl)-5-(hydroxymethyl)oxolan-3-ol), an adenosine analogue, is isolated from various natural plant species, such as *Cordyceps sinensis*, *Cordyceps militaris*, and *Ophiocordyceps sinensis*, and has multifarious physiological actions, including antioxidative, antiproliferative, antitumor, antimetastatic, immune-enhancing, and proapoptotic activities [106, 107]. More recently, a report by Dong et al. indicated that cordycepin consumption dose-dependently suppresses the proliferation of breast cancer cells in vitro following *γ*-rays exposure and reinforced therapeutic effects after radiation therapy in vivo. This is associated with promoting apoptosis and decreasing protein levels of Nrf2 and target gene HO-1, thus increasing intracellular ROS levels [91]. Cordycepin was also found to enhance the killing capability of RT against other tumors such as oral cancer and cervical cancer [108, 109].

### 4.5. Berberine (BBR)

BBR (16,17-dimethoxy-5,7-dioxa-13-azoniapentacyclo[11.8.0.02,10.04,8.015,20]henicosa-1(13),2,4(8),9,14,16,18,20-octaene), which has the characteristic of low toxicity, is a natural small molecule quaternary ammonium salt compound. It is an isoquinoline alkaloid exists in traditional Chinese medicine *Coptis chinensis* and *Hydrastis canadensis* as well as other *Berberis* plants; it has a wide spectrum of biological properties, including anticancer, anti-inflammatory, antiproliferative, antiviral, antioxidative, and antiapoptotic activities [110–112]. A scientific paper revealed that BBR cooperated with X-ray irradiation to inhibit Huh7 and HepG2 cells proliferation by downregulation of Nrf2 and target protein NQO1and HO-1 [16]. Meanwhile, Huh7 cells xenograft studies discovered that BBR combined with 8 Gy X-irradiation could also effectively repress tumor cells growth [16]. In addition, BBR provides RT amplified cytotoxicity against several cancers including osteosarcoma, esophagus carcinoma, nasopharyngeal, and prostate cancers [113–116].

### 4.6. Diallyl Disulfide (DADS)

DADS (3-(prop-2-enyldisulfanyl)prop-1-ene), also referred to as garlicin, is an oil-soluble organic diallyl polysulfide extracted from the annual bulbous herbaceous plant Garlic (*Allium sativum* L.), which is generally used as spices or used in the treatment or prevention of a host of human ailments, especially infectious diseases and cancer [117, 118]. Recently, investigators revealed that pretreatment with 40 *μ*M DADS for 24 h, followed by X-irradiation, was capable of hindering A549 cells proliferation, migration, and invasion, while modulating the epithelial-mesenchymal transition (EMT)-related proteins including E-cadherin and N-cadherin, and these effects were related to the decreasing levels of Nrf2 and Nrf2-driven antioxidant molecules NQO1 and HO-1 [15]. Besides, DADS could enhance radiosensitivity of cervical cancer HeLa cells to carbon ion beams [119].

### 4.7. Brusatol (BRU)

BRU (methyl (1R,2S,3R,6R,8R,13S,14R,15R,16S,17S)-10,15,16-trihydroxy-9,13-dimethyl-3-(3-methylbut-2-enoyloxy)-4,11-dioxo-5,18-dioxapentacyclo[12.5.0.01,6.02,17.08,13]nonadec-9-ene-17-carboxylate), the most potent and selective Nrf2 inhibitor, is a cytotoxic quassinoid natural extract from *Brucea javanica* and is frequently employed as a traditional Chinese herbal medicine for the treatment of amebiasis, cancer, and malaria [120–122]. Sun et al. investigated the radiosensitizing effects of BRU on the growth of A549 nonsmall cell lung cancer (NSCLC) cells in vitro, showing that BRU plus 6 Gy *γ*-rays could synergistically reduce the viability of A549 cells and promote cell death, via inducing ROS production and DNA fragmentation, which was attributed to interfering with the expression of Nrf2 protein [92]. Another report suggested that BRU and 8 Gy of gamma radiation induce Nrf2-dependent ataxia telangiectasia mutations and the Rad3-related kinase (ATR)-checkpoint kinase 1 (CHK1) pathway [93].

### 4.8. Alpinumisoflavone (AIF)

AIF (5-hydroxy-7-(4-hydroxyphenyl)-2,2-dimethylpyrano[3,2-g]chromen-6-one), a high water-insoluble plant-derived prenylated isoflavonoid compound, originates from the medicinal plants such as *Derris eriocarpa* and *Cudrania tricuspidata*, which are commonly utilized worldwide in osteoprotection, antioxidation, anti-inflammation, antibacteria, antimetastasis, antiatherosclerosis, and neuroprotection [123, 124]. Robust results disclosed that 5 *μ*M AIF in combination with 6 Gy X-rays exposure displayed significantly suppression of cell proliferation in Eca109 and KYSE30 cells, as well as markedly elevating the proportion of *γ*-H2AX foci, G2/M phase arrest and apoptotic cells, owing to downregulating Nrf2 and downstream effectors NQO1 and HO-1 [94]. Eca109 cells xenograft experiment also showed that AIF strengthen sensitivity of esophageal squamous cell carcinoma to RT thorough inducing ROS formation in an Nrf2-dependent manner [94].

### 4.9. Ferulic Acid (FA)

FA ((E)-3-(4-hydroxy-3-methoxyphenyl)prop-2-enoic acid), one of the most common simply natural phenolic acids, contains free radicals as an electron donors and exists widely in diverse edible plants and medicinal herbs, such as *Triticum aestivum* L. and *Oryza sativa* L. FA is a low-toxic natural product and has numerous pharmacological impacts including antioxidant, anti-inflammatory, antioncogenic, cardioprotective, neuroprotective, and enzyme-regulating effects [125, 126]. Das et al. showed that incubation with 90 *μ*M FA for 3 h, followed by a dosage of 8 Gy *γ*-rays or treatment with 100 *μ*M FA for 6 h before exposure to 7.5 Gy *γ*-irradiation, was able to remarkably destroy A549 lung cancer cells and hepatocytic carcinoma HepG2 cells, respectively [95]. Scientific article indicated that the combination therapy regimen could promote ROS accumulation, enhance oxidative stress, and induce apoptosis as well as block mitosis in G2/M phase in both tumor cells, partially due to repression of Nrf2 and Nrf2-dependent effectors [95]. Additionally, FA dramatically abbreviated the resistance of CT26 cells to *γ*-rays in vivo, too [95].

### 4.10. Epigallocatechin-3-Gallate (EGCG)

Green tea is one of the most consumed beverages worldwide, which derives from the leaves of the tea plant *Camellia sinensis*. The main bioactive polyphenol component, catechins, has broad health benefits in chronic diseases such as cardiovascular diseases, diabetes, obesity, and various cancers [127]. EGCG ([(2R,3R)-5,7-dihydroxy-2-(3,4,5-trihydroxyphenyl)-3,4-dihydro-2H-chromen-3-yl] 3,4,5-trihydroxybenzoate), as the main catechin compound, is the most abundant in dried green tea leaves, with poor absorption and pluripotency. Among the numerous functions, the chemopreventive and therapeutic activities of malignant tumors have attracted great interest of researchers [128]. Laboratory studies have confirmed synergistic antitumor effects of EGCG and radiation against leucocythemia, hepatoma, nasopharyngeal, and colorectal carcinoma [96, 129–131]. Specifically, for example, 12.5 *μ*M EGCG supplemented with 2 Gy X-rays irradiation significantly restrained HCT-116 cells survival via supporting the expression of autophagy-related gene microtubule-associated protein light chain 3 (LC3) and the apoptotic gene caspase-9 caused by Nrf2 upregulation [96].

## 5. Potential m6A Modification of Nrf2 Modulated by Dietary Phytochemicals

The expression of Nrf2 is strictly regulated by genetic and epigenetic mechanisms. Especially in recent years, the epigenetic regulation of Nrf2 in tumorigenesis has gained increasing attention, such as the alterations in DNA/RNA methylation patterns, histone modifications, and modulation of noncoding RNAs [132]. RNA modification is one of the most important epigenetic modifications in posttranscriptional regulation, and N6-methyladenosine (m6A) RNA methylation has been implicated in multiple biological processes, including radioresistance of cancer cells [133]. In addition, abnormalities in m6A RNA methylation regulatory proteins, such as the change of “writers” like methyltransferase-like protein 3/14 (METTL3/14), “erasers” like fat mass and obesity-associated protein (FTO) and AlkB homolog 5 (ALKBH5), and “readers” like YT521-B homology (YTH) domain family protein 1-3 (YTHDF1-3) and YTH domain-containing protein 2 (YTHDC2), are also reported to be involved in the radioresistance of cancer cells [134].

**Figure 6:**
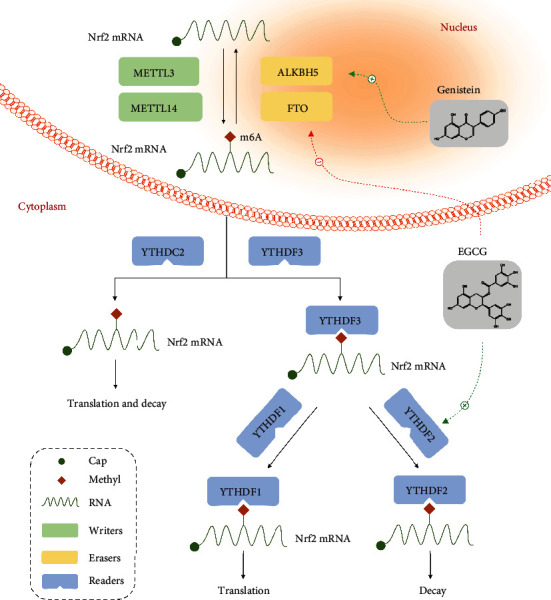
Potential mechanisms underlying m6A modification of Nrf2 mRNA and targets of dietary phytochemicals. The m6A RNA methylation of Nrf2 is conducted by methyltransferases (writers), demethylases (erasers), and m6A-binding proteins (readers). “Writers” like METTL3/14 could catalyze m6A modification on Nrf2 mRNA. “Erasers,” including FTO and ALKBH5, could remove m6A bases. “Readers,” such as YTHDF1-3 and YTHDC2, could recognize the sites modified by m6A to mediate diverse functions such as translation and RNA degradation. In addition, the expression of Nrf2 is regulated via m6A methylation, which is accomplished by modulation of “erasers” or “readers” by the dietary phytochemical compounds, EGCG and genistein.

Dietary phytochemicals have been demonstrated to have the potential to regulate Nrf2 expression by modulating m6A modification. As shown in Figure 6, m6A-Nrf2 RNA methylation was related to upregulation of YTHDF1-3 and YTHDC2 and downregulation of ALKBH5 and FTO in HepG2 human hepatoma cells [135]. Furthermore, EGCG could inhibit the generation of FTO and promote the production of YTHDF2 in 3T3-L1 cells [136], and genistein could facilitate the expression of “eraser,” ALKBH5, in mice kidney [137]. Herein, we raise a hypothesis that EGCG and genistein might also regulate m6A-Nrf2 level to influence Nrf2 expression, thus enhancing the sensitivity of cancer cells to RT, but further investigations are needed. Moreover, whether the regulation of Nrf2 by other natural radiosensitizers is mediated by regulating m6A modification still warrants exploration.

## 6. Conclusions

Overall, previous preclinical studies have provided a great abundance of natural Nrf2 inhibitors, which have radiosensitive effects on various cancers. However, there are still a few dietary phytochemicals, such as EGCG, that could activate Nrf2 and have a potent ability to enhance radiosensitivity of cancer cells. Since EGCG also has been announced as a major Nrf2 activator in other studies [138, 139], it is worthwhile to further investigate the Nrf2-modulating intrinsic mechanisms of radiosensitization by EGCG. Moreover, none of these natural radiosensitizers targeting Nrf2 mentioned above yielded strong practicable results, so researchers should pay attention to their development in clinical application. Additionally, we still ought to focus on the genotoxicity of these phytochemicals, although most of them are characterized by low toxicity. Owing to the poor bioavailability of most of these natural compounds, such as ISL [140], cordycepin [141], BRU [142], DADS [143], BBR [144], FA [145], and EGCG [146], which limits their clinical application, new technologies and advanced approaches are needed to improve the situation. A promising approach to address low bioavailability and systemic toxicity is the application of drug-loaded nanodrug delivery systems, including microemulsions, nanoemulsions, emulsions, nanoparticles, liposomes, biopolymer microgels, dendrimers, and micelles [147, 148]. Recently, several strategies have been developed to enhance the bioavailability of these phytochemicals, for instance, DADS-loaded solid lipid nanoparticles [147], cordycepin phycocyanin-based micelles [148], BBR hyaluronate liposomes [149], BRU-loaded self-microemulsions [142], EGCG-encapsulated nanoliposomes [150], AIF-loaded polymeric micelles [123], and ISL-loaded self-microemulsions [151]. These novel delivery systems could enhance the bioavailability and targeting characteristic and exhibits a superior pharmacokinetic profile.

On one hand, however, in cancer cells, Nrf2 inhibition confers radiosensitizing effects through inhibiting ROS scavenging, promoting DNA damage as well as reducing the expression of downstream target protein including NQO1 and HO-1 or cross-talk with other signal molecules like HIF-1 and NF-*κ*B. On the other hand, in normal cells, downregulation of Nrf2 may induce radiotoxicity, thereby strengthening radiation-induced injury. Therefore, the dual roles of Nrf2 inhibition should be taken into consideration in cancer therapy. And as mentioned above, the nanotechnology-based drug delivery systems are the most encouraging means to enhance the tumor-targeting ability of these phytochemicals and could reduce damage to normal tissues. Besides, structural modifications [152] and antibody-drug conjugates (ADCs) [153] also have been widely used to selectively deliver chemical drugs directly to the target cancer cells, thus decreasing drug toxicity and improving drug bioavailability.

Finally, Nrf2 could be regulated by multiple signal molecules, such as PKC, Akt, and ERK. And a number of phytochemicals, such as ferulic acid, apigenin, and baicalein, have been shown to modulate Nrf2 expression in doxorubicin-induced cardiotoxicity [154] or other diseases [155] by affecting these upstream proteins. Consequently, these studies proved us a possible orientation to discover other natural substances that could inhibit Nrf2 or design novel Nrf2 inhibitors, via indirectly inhibiting its upstream targets. Moreover, we speculated that dietary phytochemicals may regulate m6A modification to affect Nrf2 levels based on current evidence, and it might be a better strategy to explore the indirect means in Nrf2 regulation, such as the modulation of noncoding RNAs including microRNAs (miRNAs), circular RNAs (circRNAs), and long-coding RNAs (lncRNAs), or other epigenetic-related enzymes. Thus, more exhaustive regulatory roles of dietary phytochemicals on Nrf2 deserve further investigated to elucidate their pharmacological effects.

## Figures and Tables

**Table 1:** Studies on dietary phytochemicals with potential radiosensitizing activities by modulating Nrf2.

Name	Source	Cancer type	IR	Treatment *in vivo*	Treatment *in vitro*	Mechanisms	Effects	Refs.
ISL	Licorice	Human liver cancer HepG2 cells	X-rays (4 Gy)	10 mg/kg for 6 h	10 *μ*g/ml for 6 h	↓Protein and nuclear import of Nrf2; ↑Nox2 protein	↓Cell proliferation; ↑cell apoptosis; ↑ROS; ↑oxidative damage; ↑DNA damage; ↓tumor growth	[87, 88]
Genistein	Soybean	Human nonsmall cell lung carcinoma A549 cells	X-rays (4 Gy)	NA	10 *μ*M for 48 h	↓Nuclear import of Nrf2; ↑mRNA of Keap1	↓Cell growth; ↑oxidative damage; ↑ROS; ↑ratio of GSH/ GSSG; ↑apoptosis; ↓NQO1; ↓HO-1	[89]
CGN	Giant milkweed	Human lung cancer A549, NCI-H460 and NCI-H446 cells	X-rays (1-6 Gy)	NA	0.5 or 1 *μ*M for 24 h	↓Protein of Nrf2	↓Surviving fraction; ↑DNA damage; ↑ROS; ↑oxidative damage; ↑G2/M phase arrest; ↓NQO1; ↓TrxR1; ↓HO-1	[90]
Cordycepin	Chinese caterpillar fungus	Human breast cancer MCF-7 and MDA-MB-231 cells	*γ*-rays (2-6 Gy)	Postirradiated model: 30 mg/kg; preirradiated model: 32 *μ*M	32-256 *μ*M for 2 h	↓Protein and mRNA of Nrf2	↓Cell proliferation; ↑G2/M phase arrest; ↑apoptosis; ↑ROS; ↑DNA damage; ↓HO-1; ↓tumor growth	[91]
BBR	Chinese goldthread	Human hepatoma Huh7 and HepG2 cells	*γ*-rays (2-6 Gy)	5 mg/kg for 25 days combined with 8 Gy radiation	10-40 *μ*M for 24 h n	↓Nuclear import of Nrf2	↓Surviving fraction; ↑G0/G1 phase arrest; ↑apoptosis; ↑ROS; ↑oxidative damage; ↑SOD; ↑GPx; ↓NQO1; ↓HO-1; ↓tumor growth	[16]
DADS	Garlic	Human nonsmall cell lung carcinoma A549 cells	X-rays (2-8 Gy)	NA	40 *μ*M for 24 h	↓Protein and mRNA of Nrf2	↓Cell viability; ↓cell proliferation; ↓colony formation; ↓cell migration; ↓cell invasion; ↓EMT; ↓MMP-2; ↓MMP-9; ↓NQO1; ↓HO-1	[15]
BRU	Fructus bruceae	Human non-small cell lung cancer A549 cells	*γ*-rays (2-10 Gy)	2 mg/kg for 24 h	80 nM for 4 h	↓Protein and nuclear import of Nrf2	↓Cell viability; ↓surviving fraction; ↑ROS; ↑DNA damage; ↓p-CHK1; ↓p-ATR; ↑apoptosis; ↓tumor growth	[92, 93]
AIF	Mandarin melon berry	Human esophageal squamous cell cancer Eca109 and KYSE30 cells	X-rays (2-6 Gy)	20 mg/kg for 10 days	5 *μ*M for 4 or 24 h	↓The protein expression of Nrf2	↑DNA damage; ↑apoptosis; ↑G2/M phase arrest; ↓tumor growth; ↑ROS; ↓NQO1; ↓HO-1	[94]
FA	Tomatoes, wheat bran, cucurbit, orange	Human liver carcinoma HepG2 cells, human nonsmall cell lung cancer A549 cells, and mouse colon carcinoma cells CT26 cells	*γ*-rays (7.5-15 Gy)	50 mg/kg every other day for 5 doses	1) 90 *μ*M for 3 h in A549 cells; 2) 100 *μ*M for 6 h in HepG2 cells; 3) 300 *μ*M for 4 h in CT26 cells	↓Nuclear translocation of Nrf2, NF-*κ*B/p65 and STAT3; ↑p38-MAPK; ↑p-Akt;	↑ROS; ↑apoptosis; ↓cell proliferation; ↓COX-2; ↓MMP-9; ↓VEGF; ↓PDGFR*β*; ↓PECAM1; ↑apoptosis; ↑DNA damage; ↑G2/M phase arrest	[95]
EGCG	Green tea	Human colon cancer HCT-116 cells	X-rays (2 Gy)	NA	12.5 *μ*M for 24 or 48 h	↑Nuclear import of Nrf2	↓Colony formation; ↓cell proliferation; ↓cell viability; ↑apoptosis; ↑autophagy	[96]

## Data Availability

The data supporting this review article are from previously reported studies and datasets, which have been cited. The processed data are available from the corresponding author upon request.
